# Multiple Antimicrobial Resistance in Plague: An Emerging Public Health Risk

**DOI:** 10.1371/journal.pone.0000309

**Published:** 2007-03-21

**Authors:** Timothy J. Welch, W. Florian Fricke, Patrick F. McDermott, David G. White, Marie-Laure Rosso, David A. Rasko, Mark K. Mammel, Mark Eppinger, M.J. Rosovitz, David Wagner, Lila Rahalison, J. Eugene LeClerc, Jeffrey M. Hinshaw, Luther E. Lindler, Thomas A. Cebula, Elisabeth Carniel, Jacques Ravel

**Affiliations:** 1 National Center for Cool and Cold Water Aquaculture, Agricultural Research Service, United States Department of Agriculture (USDA), Kearneysville, West Virginia, United States of America; 2 The Institute for Genomic Research, Rockville, Maryland, United States of America; 3 Office of Research, Center for Veterinary Medicine-Food and Drug Administration (CVM-FDA), Laurel, Maryland, United States of America; 4 Institut Pasteur, Yersinia Research Unit, Paris, France; 5 University of Texas Southwestern Medical Center at Dallas, Dallas, Texas, United States of America; 6 Office of Applied Research and Safety Assessment, Center for Food Safety and Applied Nutrition-Food and Drug Administration (CFSAN-FDA), Laurel, Maryland, United States of America; 7 Northern Arizona University, Flagstaff, Arizona, United States of America; 8 Institut Pasteur, Antananarivo, Madagascar; 9 Department of Zoology, North Carolina State University, Fletcher, North Carolina, United States of America; 10 Public Health Laboratory Services, DoD-GEIS, Silver Spring, Maryland, United States of America; Baylor College of Medicine, United States of America

## Abstract

Antimicrobial resistance in *Yersinia pestis* is rare, yet constitutes a significant international public health and biodefense threat. In 1995, the first multidrug resistant (MDR) isolate of *Y. pestis* (strain IP275) was identified, and was shown to contain a self-transmissible plasmid (pIP1202) that conferred resistance to many of the antimicrobials recommended for plague treatment and prophylaxis. Comparative analysis of the DNA sequence of *Y. pestis* plasmid pIP1202 revealed a near identical IncA/C plasmid backbone that is shared by MDR plasmids isolated from *Salmonella enterica* serotype Newport SL254 and the fish pathogen *Yersinia ruckeri* YR71. The high degree of sequence identity and gene synteny between the plasmid backbones suggests recent acquisition of these plasmids from a common ancestor. In addition, the *Y. pestis* pIP1202-like plasmid backbone was detected in numerous MDR enterobacterial pathogens isolated from retail meat samples collected between 2002 and 2005 in the United States. Plasmid-positive strains were isolated from beef, chicken, turkey and pork, and were found in samples from the following states: California, Colorado, Connecticut, Georgia, Maryland, Minnesota, New Mexico, New York and Oregon. Our studies reveal that this common plasmid backbone is broadly disseminated among MDR zoonotic pathogens associated with agriculture. This reservoir of mobile resistance determinants has the potential to disseminate to *Y. pestis* and other human and zoonotic bacterial pathogens and therefore represents a significant public health concern.

## Introduction

*Yersinia pestis*, the etiological agent of plague, is a zoonotic bacterial pathogen that has caused multiple pandemics resulting in an estimated 200 million human deaths [1]. Plague has recently been recognized as a re-emerging disease as small outbreaks continue to occur globally [2]. This reappearance, coupled with its potential for aerosol dissemination and associated high mortality rate, also makes *Y. pestis* one of the most dangerous bioterrorism agents [3]. Antimicrobial resistance in *Y. pestis* is rare but constitutes a significant public health threat given that antimicrobials are critical both for plague treatment and prevention of human-to-human transmission. Furthermore, no vaccine is currently available. Thus, multidrug resistant (MDR) *Y. pestis* would likely have a major human health impact, complicating the control of outbreaks and leading to high mortality rates. Accordingly, the isolation of a MDR *Y. pestis* strain (IP275) in 1995 caused considerable alarm in the public health and biodefense communities [4], [5]. Strain IP275 was isolated from a bubonic plague patient in Madagascar and was shown to contain a self-transmissible plasmid (pIP1202) that conferred high-level resistance to at least eight antimicrobials, including streptomycin, tetracycline, chloramphenicol and sulfonamides, drugs recommended for plague prophylaxis and therapy [3]. This is the only documented case of MDR *Y. pestis*, although resistance monitoring of this organism is not conducted systematically [4].

Recently there also has been a rapid worldwide emergence of MDR foodborne bacterial pathogens [6], [7]. While these resistant variants can be transferred to humans via contaminated food supplies, subsequent infections usually result in a self-limited gastroenteritis that does not require antimicrobial therapy [8]. However, since these MDR determinants are often encoded on mobile plasmids, the potential transfer of MDR phenotypes from foodborne pathogens to more virulent human pathogens, including *Y. pestis,* constitutes a serious public health threat.

Here we report the complete sequence and comparative analysis of three nearly identical large (>150 kb) MDR plasmids isolated from *Yersinia pestis* [5], *Salmonella enterica* ser. Newport and *Yersinia ruckeri*. Our findings indicate a very recent common origin for these plasmids. Moreover, we present evidence that a common plasmid backbone is prevalent among *E. coli*, *Klebsiella* sp. and multiple *Salmonella* serotypes isolated from retail meats in the US, and among some food animal isolates of *E. coli*. Our data imply that high levels of MDR in the causative agent of plague may rapidly evolve naturally, and present a vital biomedical, public health, and biodefense threat.

## Results and Discussion

**Figure 1:**
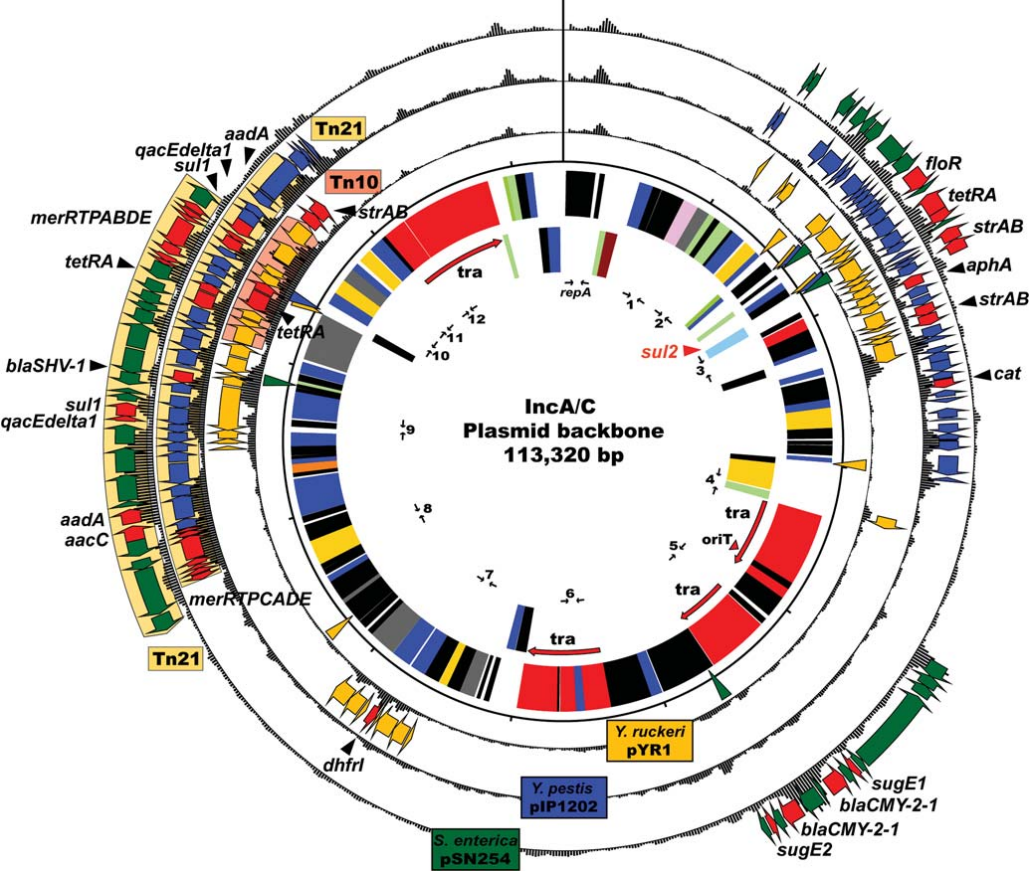
Circular representation of the IncA/C backbone (inner circle) and laterally acquired regions (outer circles) on each of the three plasmids. Nucleotide composition is represented on each of the outer circles and PCR primers used in this study are indicated in the inner circle. Antimicrobial determinants are colored red and labeled with gene names as follows: sulfonamides (*sul1, sul2*); phenicols (*cat, floR*); tetracyclines (*tetRA*); aminoglycosides/aminocyclitols (*aacC, aadA, aphA, strAB*); quaternary ammonium compounds (*qacEdelta1, sugE1, sugE2*); β-lactams (*blaCMY-2-1, blaCMY-2-2, blaSHV-1*); trimethoprim (*dhfrI*); mercury ions (*merRTPABDE, merRTPCADE*). Sequences described in this manuscript have been deposited in GenBank, accession numbers are CP000603 (pIP1202), CP000604 (pSN254), CP000602 (pYR1).

Comparative analysis of the complete DNA sequence of the *Y. pestis* pIP1202 plasmid (182,913 bp) revealed a shared IncA/C plasmid backbone of 113,320 bp with MDR plasmids pSN254 (176,473 bp) and pYR1 (158,038 bp) isolated respectively from the foodborne pathogen *Salmonella* Newport SL254 and the fish pathogen *Yersinia ruckeri* YR71 (Fig. 1). The shared backbone regions (99-100% nucleotide identity) consist of 135 syntenic genes with similar codon usage that encode functions such as plasmid replication/maintenance and type IV conjugative transfer (25 genes). A total of 85 genes have no assigned function. Notably, the backbone also includes a gene (*sul2*) conferring resistance to sulfonamides, a class of synthetic antimicrobials first introduced into clinical use in the 1930's, suggesting that these plasmids evolved from the same sulfonamide resistant ancestor. All other antimicrobial resistance determinants are integrated at four sites comprising laterally acquired DNA as shown by the deviating nucleotide composition of these regions (Fig. 1). Each plasmid also carries a laterally acquired region integrated at either side of a gene containing repetitive sequences of the RHS type [9], which may act as an integration hotspot for acquired DNA. A large fraction of antimicrobial resistance genes are encoded within either of two transposons, Tn*21* and Tn*10* (Fig. 1). A region in pSN254 contains duplicate copies of the *bla*_CMY-2_ gene. The number of resistance determinants varies with four on pYR1, ten on pIP1202 and 13 on pSN254, several of which are present in multiple copies (Table 1 and Fig. 1). In addition, both pIP1202 and pSN254 carry a mercury resistance operon, a hallmark of the transposon Tn*21*-family [10].

**Table 1:** Antimicrobial resistance determinants.

	Backbone	***Y. pestis*** ** (pIP1202)**	***S. enterica*** ** (pSN254)**	***Y. ruckeri*** ** (pYR1)**
Resistance phenotypes*	Su	ACKSSpTMSul	ACSSulTCfAxG	STTmSul
β-lactams	-	*bla_SHV-1_*	*bla*_CMY-2_ *1, bla*_CMY-2_ *2*	-
Aminoglycosides/Aminocyclitols	-	*strAB, aadA, aphA*	*strAB, aacC, aadA*	*strAB*
Tetracyclines	-	*tetRA*_class D_	*tetRA*_class A_	*tetRA*_class B_
Phenicols	-	*cat*	*floR*	-
Quaternary ammonium compounds	-	*qacEdelta1*	*qacEdelta1, sugE1, sugE2*	-
Sulfonamides	*sul2*	*sul1, sul2*	*sul1, sul2*	*sul2*
Trimethoprim	-	-	-	*dhfrI*

* Abbreviations used for antibiotics: A, ampicillin; C, chloramphenicol; S, streptomycin; Sul, sulfonamide; T, tetracycline; Cf, cephalothin, Ax, ceftriaxone; G, gentamicin; Sp, spectinomycin; M, minocycline; Tm, trimethoprim.

While MDR *Y. pestis* and *Y. ruckeri* isolates are rarely reported, monitoring of antimicrobial resistance in *Salmonella* has shown an escalating incidence of MDR strains resistant to an increasing number of drugs (i.e.≥eight antimicrobials) [11]. Moreover, *S.* Newport MDR strains harboring plasmids encoding resistance to expanded-spectrum cephalosporins (Newport MDR-AmpC phenotype) are widely disseminated within the United States and have been isolated from cattle, ground beef and ill humans [12]. As plasmid pSN254 confers the Newport MDR-AmpC resistance phenotype, we sought to determine the occurrence and distribution of the IncA/C plasmid backbone among 125 MDR *Salmonella* strains recovered from retail meats from 2002 to 2005 through the National Antimicrobial Resistance Monitoring System (NARMS see cite above), as well as a small collection of *E. coli* strains recovered from food animals and *Klebsiella* isolates from ground turkey meat from Iowa. Using the *repA* gene as marker, primary screening by PCR for the IncA/C replicon [13] revealed 70 *repA-*positive samples that were subsequently probed with a panel of twelve PCR assays targeting backbone regions (shown in Fig. 1). These findings indicated that IncA/C plasmid backbones were present in all *repA*-positive strains, in all meat types sampled, including chicken, turkey, pork and ground beef and in all ten states that participate in the NARMS retail surveillance (Table 2). Several IncA/C positive strains were found in California, New Mexico and Oregon, areas of the United States where *Y. pestis* is endemic. IncA/C backbones were detected in representatives of *S. enterica* Newport, Heidelberg, Kentucky, Dublin, Bredeney, and Typhimurium. In addition, screening of *E. coli* and *Klebsiella* MDR isolates revealed the presence of this plasmid backbone in the nine tested *Klebsiella* isolates recovered from ground turkey collected in Iowa, and *E. coli* isolated from a calf in North Dakota and a broiler chicken from Georgia (Table 2). A majority of *S.* Newport and *S*. Heidelberg strains (9/14) and several *Klebsiella* isolates (3/9) were positive for all twelve markers, while other *Salmonella* serotypes were positive for 50% or greater of the assayed loci (Table 2).

**Table 2:** Antimicrobial resistance phenotypes, IncA/C PCR profiles and conjugation frequencies of MDR NARMS isolates

Strain	Organism	State	Year	Source	Antimicrobial resistance phenotype^a^														*IncA/C* PCR profile^b^												Transfer^c^
					A	C	S	Su	T	Ti	G	K	Ag	Ax	N	Ak	Cp	Sxt													
30034	*S*. Typhimurium	CA	2003	Turkey	A	C	S	Su	T	Ti	-	-	Ag	-	N	-	-	-	1	2	3	4	5	-	-	-	-	10	11	12	-
32412	*S*. Typhimurium	CT	2003	Chicken	A	-	-	Su	T	Ti	-	-	Ag	-	-	-	-	-	1	2	3	4	5	6	7	8	9	-	11	12	8.4 x 10^-6^
32463	*S*. Typhimurium	NY	2003	Chicken	A	-	-	Su	T	Ti	-	K	Ag	-	-	-	-	-	-	2	3	4	5	6	7	8	9	-	11	12	1.1 x 10^-5^
N1577	*S*. Typhimurium	MD	2004	Chicken	A	-	-	Su	T	Ti	-	-	Ag	-	-	-	-	-	1	2	-	4	-	6	-	-	-	-	11	12	-
N1583	*S*. Typhimurium	MD	2004	Chicken	A	-	-	Su	T	Ti	-	-	Ag	-	-	-	-	-	1	2	-	4	5	-	-	-	-	-	11	12	-
N1591	*S*. Typhimurium	MD	2004	Chicken	A	-	-	Su	T	Ti	-	K	Ag	-	-	-	-	-	1	2	-	4	-	6	7	8	9	-	11	12	8.2 x 10^-7^
N145	*S*. Typhimurium	CT	2004	Chicken	A	-	-	Su	T	Ti	-	-	Ag	-	-	-	-	-	1	2	3	4	5	6	-	-	-	-	11	12	3.8 x 10^-5^
N167	*S*. Typhimurium	CT	2004	Chicken	A	-	-	Su	T	Ti	-	-	Ag	-	-	-	-	-	1	2	-	4	-	6	-	-	-	-	11	12	3.2 x 10^-6^
N509	*S*. Typhimurium	NY	2004	Chicken	A	-	-	Su	T	Ti	-	K	Ag	-	-	-	-	-	1	2	3	4	5	6	7	8	9	-	11	12	3.7 x 10^-5^
N520	*S*. Typhimurium	NY	2004	Chicken	A	-	-	Su	T	Ti	-	-	Ag	-	-	-	-	-	1	2	-	4	-	6	7	8	9	-	11	12	2.9 x 10^-6^
N168	*S*. Typhimurium	CT	2004	Chicken	A	-	-	Su	T	Ti	-	K	Ag	-	-	-	-	-	1	2	3	4	5	6	7	8	9	-	11	12	-
N169	*S*. Typhimurium	CT	2004	Chicken	A	-	-	Su	T	Ti	-	-	Ag	-	-	-	-	-	1	2	3	4	5	6	7	8	9	-	11	12	-
N176	*S*. Typhimurium	CT	2004	Chicken	A	C	S	Su	T	Ti	-	-	Ag	-	-	-	-	-	1	2	3	4	5	6	-	-	-	-	11	-	-
N177	*S*. Typhimurium	CT	2004	Chicken	A	-	-	Su	T	Ti	-	-	Ag	-	-	-	-	-	1	2	3	4	5	6	7	8	9	-	11	12	-
N499	*S*. Typhimurium	NY	2004	Chicken	A	-	-	Su	T	Ti	-	K	Ag	-	-	-	-	-	1	2	3	4	5	6	7	8	9	-	11	12	-
N1563	*S*. Typhimurium	MD	2004	Chicken	A	-	-	Su	T	Ti	-	K	Ag	-	-	-	-	-	1	2	3	4	5	6	7	8	9	-	11	12	9.0 x 10^-6^
N1575	*S*. Typhimurium	MD	2004	Chicken	A	-	-	Su	T	Ti	-	-	Ag	-	-	-	-	-	1	2	3	4	5	6	7	8	9	-	11	12	8.4 x 10^-4^
N4528	*S*. Typhimurium	CT	2005	Chicken	A	-	-	Su	T	Ti	-	K	Ag	-	-	-	-	-	1	2	3	4	-	-	-	8	9	-	11	12	-
N4546	*S*. Typhimurium	CT	2005	Chicken	A	-	-	Su	T	Ti	-	-	Ag	-	-	-	-	-	1	2	3	4	-	-	-	8	9	-	11	12	-
N7313	*S*. Typhimurium	CT	2005	Chicken	A	-	-	Su	T	Ti	-	-	Ag	-	-	-	-	-	1	2	3	4	-	-	-	8	9	-	11	12	-
N6424	*S*. Typhimurium	NY	2005	Chicken	A	-	-	Su	T	Ti	-	K	Ag	-	-	-	-	-	1	2	3	4	5	6	-	8	9	-	11	12	2.5 x 10^-4^
N6426	*S*. Typhimurium	NY	2005	Chicken	A	-	-	Su	T	Ti	-	-	Ag	-	-	-	-	-	1	2	3	4	5	6	7	8	9	-	-	12	-
N6430	*S*. Typhimurium	NY	2005	Chicken	A	-	-	Su	T	Ti	-	K	Ag	-	-	-	-	-	1	2	3	4	-	6	-	8	9	-	11	12	-
N6437	*S*. Typhimurium	NY	2005	Chicken	A	-	-	Su	T	Ti	-	-	Ag	-	-	-	-	-	1	2	3	4	5	6	-	8	9	-	11	12	4.3 x 10^-3^
N5370	*S*. Typhimurium	MD	2005	Chicken	A	-	-	Su	T	Ti	-	-	Ag	-	-	-	-	-	-	2	3	4	5	6	-	8	9	-	11	12	-
N5379	*S*. Typhimurium	MD	2005	Chicken	A	-	-	Su	T	Ti	-	-	Ag	-	-	-	-	-	1	2	3	4	5	6	-	-	-	-	-	-	6.3 x 10^-5^
N5380	*S*. Typhimurium	MD	2005	Chicken	A	-	-	Su	T	Ti	-	K	Ag	-	-	-	-	-	1	2	3	4	5	6	-	8	9	-	11	12	-
N5385	*S*. Typhimurium	MD	2005	Chicken	A	-	-	Su	T	Ti	-	K	Ag	-	-	-	-	-	1	2	3	4	-	6	7	8	9	-	11	12	1 x 10^-6^
22404	*S*. Newport	CT	2002	Beef	A	-	S	Su	T	Ti	-	-	Ag	-	-	-	-	-	1	2	3	4	5	6	-	-	-	10	11	12	-
22697	*S*. Newport	MD	2002	Turkey	A	C	S	Su	T	Ti	-	-	Ag	-	-	-	-	Sxt	1	2	3	4	5	6	7	8	9	10	11	12	-
22699	*S*. Newport	MD	2002	Pork	A	C	S	Su	T	Ti	-	-	Ag	-	-	-	-	Sxt	1	2	3	4	5	6	7	8	9	10	11	12	-
22707	*S*. Newport	MD	2002	Beef	A	C	S	Su	T	Ti	-	-	Ag	-	-	-	-	-	1	2	3	4	5	6	-	-	-	10	11	12	-
29461	*S*. Newport	CA	2003	Beef	A	C	S	Su	T	Ti	-	-	Ag	-	-	-	-	-	1	2	3	4	5	6	7	8	9	10	11	12	-
29768	*S*. Newport	OR	2003	Pork	A	C	S	Su	T	Ti	-	-	Ag	-	-	-	-	-	1	2	3	4	5	6	7	8	9	10	11	12	-
N1543	*S*. Newport	GA	2004	Beef	A	C	S	Su	T	Ti	-	-	Ag	Ax	-	-	-	-	1	2	3	4	5	6	-	-	-	10	11	12	-
N635	*S*. Newport	OR	2004	Beef	A	C	S	Su	T	Ti	-	-	Ag	-	-	-	-	Sxt	1	2	3	4	5	6	7	8		10	11	12	-
N175	*S*. Kentucky	CT	2004	Chicken	A	C	S	Su	T	Ti	-	-	Ag	-	-	-	-	-	1	2	-	4	-	6	-	-	-	-	11	-	8.3 x 10^-5^
N6427	*S*. Kentucky	NY	2005	Chicken	A	-	S	-	T	Ti	-	-	Ag	-	-	-	-	-	1	2	3	4	5	6	7	8	9	-	11	12	-
N4534	*S*. I*.4,5,12:n.mot.*	CT	2005	Chicken	A	-	-	Su	T	Ti	-	-	Ag	-	-	-	-	-	1	2	3	4	5	6	7	8	9	-	11	12	-
N6312	*S*. I*.3,10 :n.mot.*	MN	2005	Turkey	A	-	S	Su	T	Ti	-	K	Ag	-	-	-	-	-	1	2	3	4	5	6	-	8	9	-	11	12	5.2 x 10^-5^
N5389	*S*. I*. 4,12 : r :-*	MD	2005	Turkey	A	C	S	Su	T	Ti	-	-	Ag	-	-	-	-	Sxt	1	2	3	4	5	-	-	-	-	10	11	12	-
20592	*S*. Heidelberg	CT	2002	Turkey	A	-	S	Su	T	Ti	-	-	Ag	-	-	-	-	-	1	2	3	4	5	-	-	-	-	10	11	12	-
21380	*S*. Heidelberg	CT	2002	Turkey	A	-	S	Su	T	Ti	G	K	Ag	-	-	-	-	-	1	2	3	4	5	6	7	8	9	10	11	12	3.3 x 10^-2^
N416	*S*. Heidelberg	NM	2004	Turkey	A	C	S	Su	T	Ti	G	-	Ag	-	-	-	-	-	1	2	3	4	5	6	7	8	9	10	11	12	9 x 10^-4^
N418	*S*. Heidelberg	NM	2004	Turkey	A	C	S	Su	T	Ti	G	K	Ag	-	-	-	-	-	1	2	3	4	5	6	7	8	9	10	11	12	4.4 x 10^-2^
N633	*S*. Heidelberg	OR	2004	Chicken	A	C	S	Su	T	Ti	-	-	Ag	-	-	-	-	-	1	2	3	4	5	6	7	8	9	10	11	12	6.3 x 10^-2^
N4504	*S*. Heidelberg	CO	2005	Turkey	A	-	-	-	-	Ti	-	-	Ag	-	-	-	-	-	1	2	3	4	5	6	7	8	9	10	11	12	-
29440	*S*. Dublin	MN	2003	Beef	A	C	S	Su	T	Ti	-	-	Ag	Ax	-	-	-	-	1	2	3	4	5	-	-	-	-	-	-	-	-
23742	*S*. Dublin	OR	2003	Beef	A	C	S	Su	T	Ti	-	-	Ag	-	-	-	-	Sxt	1	2	3	4	5	6	7	-	-	10	11	12	-
N187	S. Bredeney	CT	2004	Turkey	A	-	-	Su	T	Ti	G	-	Ag	-	-	-	-	-	1	2	3	4	5	-	-	-	-	10	11	12	-
N6328	*S*. Bredeney	MN	2005	Turkey	A	-	S	Su	T	Ti	-	-	Ag	Ax	-	-	-	-	1	2	3	4	5	-	-	-	-	10	11	12	-
10688	*Klebsiella sp.*	IA	2002	Turkey	A	C	S	Su	T	Ti	G	K	Ag	-	N	-	-	-	1	2	3	4	5	6	-	8	9	10	11	12	4.9 x 10^-3^
10689	*Klebsiella sp.*	IA	2002	Turkey	A	C	S	Su	T	Ti	G	K	Ag	-	N	-	-	-	1	-	3	-	5	-	-	8	9	10	11	-	9.3 x 10^-3^
10698	*Klebsiella sp.*	IA	2002	Turkey	A	C	S	Su	T	Ti	G	K	Ag	-	-	-	-	-	1	-	-	-	5	-	-	-	-	10	11	-	-
10978	*Klebsiella sp.*	IA	2002	Turkey	A	C	S	Su	T	Ti	G	K	Ag	-	-	-	-	-	1	2	3	4	5	6	7	8	9	10	11	12	1 x 10^-2^
11226	*Klebsiella sp.*	IA	2002	Turkey	A	C	S	Su	T	Ti	G	K	Ag	-	-	-	-	-	1	-	3	-	5	-	-	8	9	10	11	12	2.5 x 10^-3^
11228	*Klebsiella sp.*	IA	2002	Turkey	A	C	S	Su	T	Ti	G	K	Ag	-	-	-	-	-	1	-	3	-	5	-	-	8	9	10	11	12	1.2 x 10^-3^
12905	*Klebsiella sp.*	IA	2002	Turkey	A	C	S	Su	T	Ti	G	K	Ag	-	-	-	-	-	1	2	3	4	5	-	-	-	-	10	11	12	-
12906	*Klebsiella sp.*	IA	2002	Turkey	A	C	S	Su	T	Ti	G	K	Ag	-	-	-	-	-	1	-	3	-	5	-	-	-	-	10	11	12	-
13280	*Klebsiella sp.*	IA	2002	Turkey	A	C	S	Su	T	Ti	G	K	Ag	-	-	-	-	-	1	2	3	4	5	6	7	8	9	10	11	12	7.9 x 10^-4^
3311b	*E. coli*	ND	1997	Calf	A	C	S	Su	T	Ti	G	K	Ag	Ax	N	Cp	-	-	1	-	3	-	5	-	-	8	9	10	11	12	3.6 x 10^-4^
19811	*E. coli*	MD	2002	Chicken	A	C	S	Su	T	Ti	G	K	Ag	-	-	-	-	-	1	2	3	4	5	6	7	8	9	10	11	-	1.2 x 10^-2^
4420	*E. coli*	GA	2002	Chicken	A	-	S	-	-	Ti	G	K	Ag	Ax	N	-	Ak	-	1	-	3	-	5	-	-	8	9	10	11	12	4.5 x 10^-3^
32645	*E. coli*	GA	2003	Turkey	A	C	S	Su	T	Ti	G	K	Ag	-	N	-	-	-	1	-	3	4	5	6	7	8	9	10	11	-	3.3 x 10^-2^
32751	*E. coli*	GA	2003	Pork	A	C	S	Su	T	Ti	-	K	Ag	-	-	-	-	-	1	-	3	-	5	6	-	-	-	10	11	-	-
N820	*E. coli*	OR	2004	Beef	A	C	S	Su	T	Ti	G	-	Ag	-	-	-	-	-	1	2	3	4	5	-	-	-	-	10	11	-	-
N935	*E. coli*	OR	2004	Chicken	A	C	S	Su	T	Ti	-	-	Ag	-	-	-	-	-	1	-	3	-	5	-	-	-	-	10	11	-	-
N1004	*E. coli*	OR	2004	Pork	A	C	S	Su	T	Ti	-	-	Ag	-	-	-	-	-	1	2	3	4	5	-	-	-	9	10	11	-	-
N2024	*E. coli*	GA	2004	Beef	A	C	S	Su	T	Ti	-	-	Ag	-	-	-	-	-	1	2	3	-	5	-	-	-	-	10	11	-	-
N3878	*E. coli*	TN	2004	Chicken	A	C	S	Su	T	Ti	G	-	Ag	-	-	-	-	-	1	2	3	-	-	-	7	8	9	-	11	-	7.2 x 10^-3^

a amikacin (Ak), amoxicillin/clavulanate (Ag), ampicillin (A), cefoxitin (Cx), ceftiofur (Ti), ceftriaxone (Ax), chloramphenicol (C), ciprofloxacin (Cp), gentamicin (G), kanamycin (K), nalidixic acid (N), tetracycline (T), streptomycin (S), sulfamethoxazole (Su), trimethoprim/sulfamethoxazole (Sxt).

b PCR amplicons tested shown in Fig. 1.

c Estimated efficiency of transfer to Y. ruckeri; expressed as the number of transconjugants per donor; all transconjugants tested positive for the IncA/C replicon.

The discovery of these MDR IncA/C plasmids in evolutionarily distinct pathogens attests to recent genetic exchange, either directly between these bacterial species or through bacterial intermediates, and it suggests that overlap in the ecological niches of these organisms is sufficient to permit past or future plasmid transmission. This is consistent with the high identity (60 kb, 97% nucleotide identity) found between *Y. pestis* virulence plasmid pFra and *S. enterica* Typhi plasmid pHMC2 [14], [15]. The ecological location of plasmid transfer to *Y. pestis* is unknown, although it has been speculated that transfer might occur in a co-infected mammalian host or in the midgut of the flea [16], the classic vector for plague. Recent experimental evidence has shown that high frequency transfer of a streptomycin resistant R-plasmid from *E. coli* to *Y. pestis* can occur in flea midgut [17].

While identification of the exact bacterial donor or sequence of events leading to the transfer of pIP1202 to *Y. pestis ca*nnot be conclusively determined, we were able to demonstrate the transfer of the IncA/C plasmids present in the 70 MDR *repA*+foodborne enterics (Table 2) to *Y. ruckeri*. Thirty of the 70 MDR enterics tested transferred drug resistance to *Y. ruckeri* along with the IncA/C-specific *repA* marker. Interestingly, 53% (29/54) of the MDR isolates from poultry products transferred resistance to *Y. ruckeri* while only one of the 14 non-poultry isolates was transfer competent. Conjugal transfer could not be demonstrated for IncA/C plasmids from any of the *S.* Newport isolates identified, including the sequenced SL254 isolate. In these cases transfer might depend on the presence of additional transfer functions or may be limited by incompatible restriction/modification systems. In all, a large fraction of the *repA*+MDR enterics were capable of transferring resistance, suggesting that direct transfer of IncA/C MDR plasmids mediates environmental dissemination between these bacterial genera.

Our findings reveal a potential for the natural acquisition of multidrug resistance in *Y. pestis* and other MDR zoonotic bacterial pathogens. Hence, antimicrobial resistance monitoring should be expanded especially to the areas of the world where *Y. pestis* is endemic, including Asia, Africa and the Southwestern United States where both *Y. pestis* and MDR *Salmonella* have been isolated and therefore have a high probability of coming into direct contact. The plasmid detection and typing assays described here could also be used to monitor pIP1202-like antimicrobial resistance plasmids in numerous bacterial pathogens recovered from diverse environments including animal production systems, foods, and health care settings, and may be useful for the detection of these plasmids in biothreat agents.

## Materials and Methods

### Genome sequencing and Informatics

Plasmids pIP1202 and pSN254 were assembled from whole genome shotgun sequencing of *Y. pestis* IP275 and *S. enterica* serotype Newport SL254 respectively, while pYR1 was sequenced from purified plasmid DNA. Sequencing libraries were constructed as previously reported [18] and sequenced using 3730xl DNA analyzer (Applied Biosystems). Assembly and closure, followed by manual annotation were performed as previously described [18].

### PCR screening

Plasmid DNA for PCR was prepared using the QIAprep Spin Miniprep kit according to the manufacturer's procedure (Qiagen). PCR was performed using HotStarTaq (Qiagen) with the following cycling protocol: five min enzyme activation at 95°C, 26 cycles of 45 s at 95°C, 45 s at 55°C and 90 s at 72°C followed by a 10 min final extension step at 72°C. The 13 primer sets used were designed to each amplify a distinct plasmid backbone region displayed in Fig. 1. Primers used are listed in Supplemental Methods S1.

### Plasmid transfer experiments

Conjugative mating experiments were performed using a rifampicin resistant derivative of *Y. ruckeri* (YR34R) as the recipient strain. Donor (Table 2) and recipient strains were grown in an orbital shaker (225 rpm) in Brain-Heart-Infusion (BHI) medium at 37°C (donor) or 28°C (recipient) to mid-log phase. Donor and recipient bacteria were then harvested by centrifugation (6000×g, 10 min, 4°C) and 10^8^ of each were mixed and spotted onto BHI agar. Conjugation mixtures were allowed to incubate for 3 h at 28°C after which the cells were resuspended in PBS, diluted, and plated onto BHI agar containing tetracycline (20 µg/ml) for plasmid selection and rifampicin (100 µg/ml) to counter select the donor. For donor strains that did not transfer tetracycline resistance, conjugations were repeated using selection for ampicillin (100 µg/ml), ceftriaxone (8 µg/ml), chloramphenicol (100 µg/ml), and kanamycin (50µg/ml). Transconjugants were confirmed using a *Y. ruckeri*-specific bacteriophage and assayed for the presence of the *repA* marker by PCR as described above.

## Supporting Information

Methods S1 Primers used in this study
(0.03 MB DOC)
