# Supplementary material for: Multiple Antimicrobial Resistance in Plague: An Emerging Public Health Risk
Source: PLoS One. 2007 Mar 21;2(3):e309. doi: 10.1371/journal.pone.0000309 (PMC1819562; doi:10.1371/journal.pone.0000309)
Supplement: Methods S1 — Primers used in this study (0.03 MB DOC) [file pone.0000309.s001.doc]

**Supplemental Methods S1**

Primers used in this study listed 5’ to 3’:

***repA***

repAF- GAGAACCAAAGACAAAGACCTGGA

repAR- TTCTGGAGTTCGTACAGAGTGAAC

**Region 1**

R1F- AGCACGATAGCTTGTGAGTTCG

R1R- AGCAGATAAGAAGGCGATGACC

**Region 2**

R2F-CAACCCCTTACCAGCTTTGAAC

R2R- TGAGGCTGACGACAAGGTAGAG

**Region 3**

R3F- ATGCCACATGGGTAGACATCAC

R3R- GAATGCATAACGACGAGTTTGG

**Region 4**

R4F- CGTATTTCTCGTCGCTACATGC

R4R- AGTAGCGGAATCGATCCAGAAG

**Region 5**

R5F- GAACGTGCTTGATGGTTTCTTG

R5R- CTGCTCCACATGATCTACTGGG

**Region 6**

R6F- GGACGTCATCTAACCCCTGTTC

R6R- AGCAGCTCTACGCCTTTACGTC

**Region 7**

R7F- CAGCACAAACATCTTCCCAGAC

R7R- GGGTAACACCGCCAACTCTTAC

**Region 8**

R8F- GAAAGCGCAACAACACAAAGAC

R8R- TGACTACTCTTGCCAGCTTTGC

**Region 9**

R9F- GTTCAAACTCACGCTGCAAAAC

R9R- ATACCGCAGACGGAAAGAGAAG

**Region 10**

R10F- AGAATAGCCGCCGTCATAGAAG

R10R- AAAAGGCGTACCGACAAGAGAG

**Region 11**

R11F- ATCGAGGGAGTGTTCCTCTGAC

R11R- CAAGGCTGAGGGTTCCTATCAC

**Region 12**

R12F- TGCTCCAGAAAAGCAGAGTCAC

R12R- CCGGGACAAATTACAGGAGAA
